# Experimental Investigation on Centrifugal Compressor Blade Crack Classification Using the Squared Envelope Spectrum

**DOI:** 10.3390/s130912548

**Published:** 2013-09-18

**Authors:** Hongkun Li, Xuefeng Zhang, Fujian Xu

**Affiliations:** School of Mechanical Engineering, Dalian University of Technology, No. 2 Linggong Road, Dalian 116024, China; E-Mails: wenlong0118@163.com (X.Z.); xfj7811285@163.com (F.X.)

**Keywords:** centrifugal compressor, blade crack, condition classification, squared envelope spectrum

## Abstract

Centrifugal compressors are a key piece of equipment for modern production. Among the components of the centrifugal compressor, the impeller is a pivotal part as it is used to transform kinetic energy into pressure energy. Blade crack condition monitoring and classification has been broadly investigated in the industrial and academic area. In this research, a pressure pulsation (PP) sensor arranged in close vicinity to the crack area and the corresponding casing vibration signals are used to monitor blade crack information. As these signals cannot directly demonstrate the blade crack, the method employed in this research is based on the extraction of weak signal characteristics that are induced by blade cracking. A method for blade crack classification based on the signals monitored by using a squared envelope spectrum (SES) is presented. Experimental investigations on blade crack classification are carried out to verify the effectiveness of this method. The results show that it is an effective tool for blade crack classification in centrifugal compressors.

## 1. Introduction

**Figure 1.**
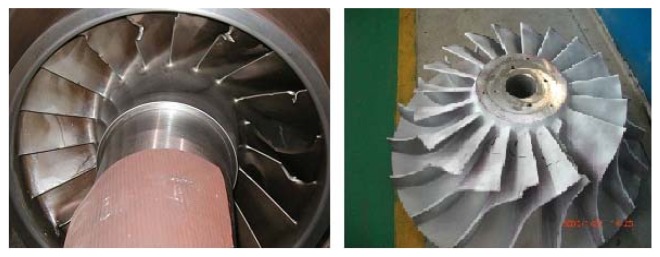
Pictures of centrifugal compressor blade cracks.

With the urgent demands for socializations, centrifugal compressors are developing to be large in scale, high in speed, and automatic in operation. As a key equipment of petrochemical factories, high efficiency and high reliability are key features of centrifugal compressors, which can produce a pressure rise through an impeller. Among the components analysis of centrifugal compressors, blades are both important and weak spots. The working conditions are affected by the fluid field, structural field, acoustic field, and high temperature field, therefore, they work under complicated conditions compared with other machines, such as gearboxes, machine tools. According to statistical analysis, 65% of centrifugal compressor malfunctions are closely related to the blades. In addition, some 40% of blade fatigue failures are not fully understood so far. Fatigue can lead to blade cracks or even failures, therefore, condition monitoring and pattern classification are important to prevent blades from failure. As the working conditions for centrifugal compressors are complex and changeable, compressors are usually operating in an off-design condition. Fluid-induced vibration is an important factor for blade fatigue failure. It contains acoustic resonance, unsteady flow, rotating stalls, and flutter [1,2]. Due to the high-velocity flow through the centrifugal compressor and impeller rotation, high-pressure fluctuations occur inside the casing. These give rise to the vibration response of the impeller. The high-pressure fluctuations can also be transferred to the centrifugal compressor casing by the inside working fluid. It works on the impeller, which leads to stress convergence and crack occurrence in the blades. Fatigue cracks are mainly caused by alternating stress. With crack growth, this can lead blade failure in the end. Catastrophes may occur in the blade fatigue failure. Investigation on alternating stress can be helpful to prevent or reduce the damage from blade cracks [3]. Examples are shown in Figure 1.

After blade crackdown, the compressor cannot function well until a new impeller is installed. This needs a long time if no backup impeller is available. For an important centrifugal compressor, this can lead to heavy losses for a factory as it may lose $500,000 a day. As well, personal safety must be considered because the breakdown of blade is dangerous as its tangential velocity can be up to 450 m/s. In the early stages of blade cracking, rigorous testing can ensure the reliable operation of a centrifugal compressor. Therefore, incipient classification of blade cracks is more and more important than ever before. Traditionally, displacement sensors are employed to monitor shaft vibration. At the same time, vibration-based condition monitoring is also used in shaft crack classification [4,5], but it is difficult to recognize shaft cracks using vibration signals. It is impossible to provide any information to characterize blade crack condition from the shaft vibration signals, making blade crack classification more difficult compared with shaft crack identification. Different methods for blade condition classification have been investigated by many researchers. Rao investigated blade crack condition classification by using characteristic vibration signal information for gas turbine blade recognition [6]. Witek investigated the experimental crack propagation for gas turbine blades via vibration signals in the laboratory but it was not in a close-loop test-rig [7]. Pressure pulsation (PP) generated by the interference between rotating blades and the stationary vanes, contains much information about the blade working conditions. This has been investigated in pump-turbine blade failure analysis [8]. Acoustic emission (AE) signals are also applied to wind turbine blade classification [9]. Support vector machines have good performance in classification and prediction. This was used in helicopter rotor blade damage detection [10]. Based on the above analysis, much investigation is also carried out on gas turbine blades, but centrifugal compressor blades are seldom investigated, despite the similarities between the two kinds of machines. Moreover, experiments on centrifugal compressor test-rigs are not investigated as there are many factors limiting the implementation, such as price, safety, classification method, monitored signals, and so on. In addition, there are no closed-loop experiments about blade crack classification.

**Figure 2.**
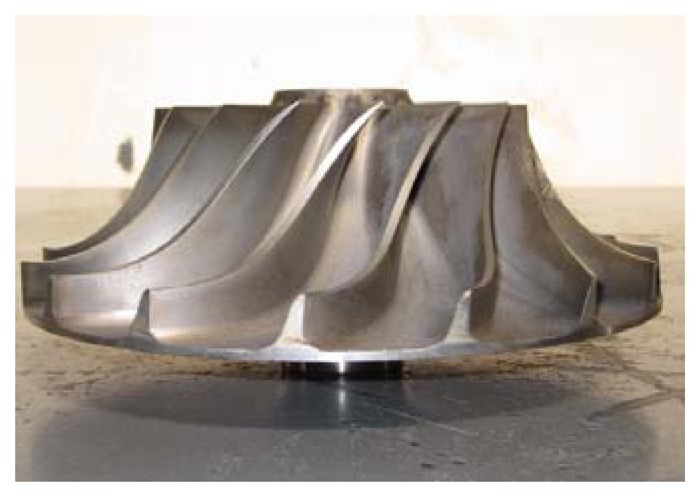
Structure of a semi-open impeller for a centrifugal compressor.

The processing technique used for centrifugal compressor impeller production is the whole milling method and the structure is shown is Figure 2. The material is stainless steel and its rigidity as compressor impeller is fine, but there are also many impeller failures as blades of centrifugal compressor are subject to centrifugal forces, alternating stresses, and stochastic loads. The variation of blade force can lead to vibrations different from normal working conditions. Then, the blade crack information can be transferred to the air and casing vibrations. As the blade structure for centrifugal compressors is different from that of gas turbines, it is important to determine the crack information as early as possible. Incipient crack classification is significant for the compressor impeller maintenance, therefore, early classification methods to alert of cracks is important to reduce losses. As the blade crack information is not monitored directly, it is also difficult to determine the crack information with traditional methods. A convenient and simple method is for centrifugal compressor blade condition monitoring and fault classification is therefore urgently needed.

If there is a crack in the blade, it leads to abnormal blade vibrations. As the blade vibration signal is difficult to obtain, other information can be used indirectly for crack pattern recognition. The differences in blade vibration are a typical characteristic when there is a crack in a centrifugal compressor blade. As an impeller rotates, air inside centrifugal compressor will glide on the blades resulting in a dynamic pressure difference. Therefore, the high-pressure pulsation signal contains blade vibration information. This also makes the PP for a cracked blade different from a normal one. Since air is the communication medium between the blade and the casing, it can deliver blade vibration information to the casing. The casing will also respond to blade vibrations because the distance between blade and casing is small. As the casing vibration is the combination of harmonic and random excitation by the rotor and high-pressure pulsation, the vibration information is complicated compared with a PP signal. At the same time, the information obtained by indirect monitoring methods is weak. Only a suitably positioned vibration sensor can determine the crack information [11]. This is also the reason why vibration signals can be applied to compressor blade crack classification. Therefore, PP and casing vibration can provide the crack information for centrifugal compressor. As the information is weak, the frequency domain information is more important for blade classification. Blade vibration frequency with a crack is different from normal working conditions. The frequency corresponding to abnormal blade vibrations will be modulated to high frequency. Blade passing frequency (BPF) usually corresponds to the high frequency. However, the crack information either in PP or casing vibration signals is weak, so it is difficult for use pattern recognition just according to time or frequency information, especially for the incipient blade crack condition. Further feature extraction methods are urgently needed for better information gathering.

The envelope technique is a powerful tool in fault classification that has been widely applied on rolling element bearings and gearbox fault diagnosis as it can demodulate the characteristic frequency (CF) from the modulated signal. However, it cannot determine the CF information if there is strong noise interference, so weak information determination methods should be investigated. The squared envelope spectrum (SES) belongs to the cyclostationary analysis families provided by Antoni [12]. It can be looked at as an improvement of the envelope technique. Borghesani investigated this method and extended it for variable operating condition classification [13]. As mentioned above, the blade crack vibration generates a CF which can be modulated to BPF. Therefore, SES can be applied to CF determination of blade cracks although there is noise interference in the practical working centrifugal compressor.

In this research, PP and the corresponding vibration signals are used for blade working condition classification by using SES. Experiments are carried out to verify the effectiveness of this method in a test-rig. According to the investigation, it can contribute to blade crack condition monitoring and early fault classification. The structure of this paper is as follows: Section 2 introduces the theory of feature extraction for blade crack classification. Section 3 describes our experimental setup for blade crack monitoring. Section 4 demonstrates PP and vibration signals analysis for blade condition classification. Concluding remarks are given in Section 5.

## 2. Theory and Method

### 2.1. Squared Envelope Spectrum

**Figure 3.**
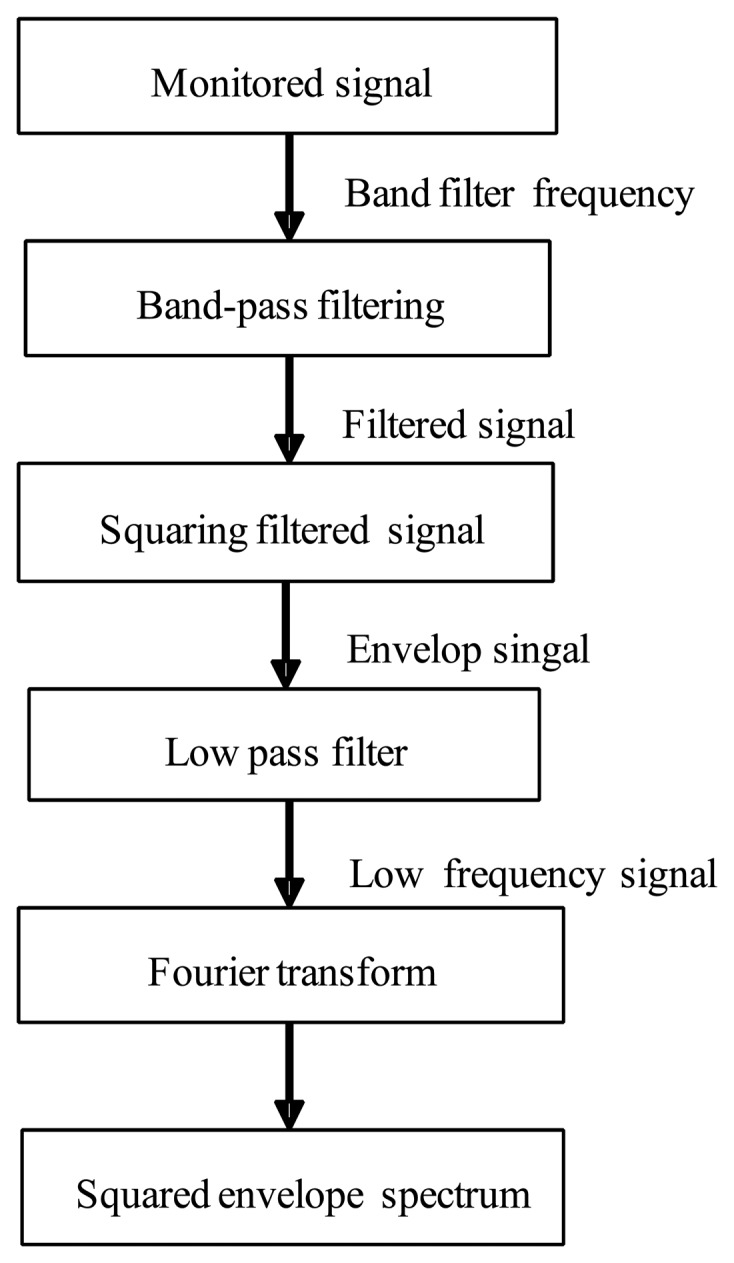
Flowchart of squared envelope spectrum analysis.

Sideband frequency analysis is helpful to determine blade cracks [1]. As there is much noise interference for monitored signals, the CF determination method must be investigated for practical condition applications. Envelope spectrum analysis is one of the most popular techniques which has been widely used in feature extraction and pattern recognition. Envelope analysis can be looked at as a cyclostationary tool to demodulate signal and determine CF according to the monitoring signal [13]. It has been applied in gearbox and rolling element bearing fault diagnosis, but it is not effective when the information in the signal is weak. SES can be viewed as a development and improvement for envelope analysis. Usually, it consists of four steps: (1) determination of the analysis frequency band; (2) design of a band-pass filter; (3) calculation of the squared band-passed signal; (4) derivation of the Fourier spectrum for the envelope signal. The SES application process is shown in Figure 3.

SES has good performance on second order cyclostationary characteristic information determination [14,15]. For a signal *x*(*t*), its filtered signal can be shown as Equation (1)

(1) $$x_{\text{filter}}(t)=x(t)⊗\text{filter}(t)$$

Therefore, the SES of filtered signal *x*(*t*) can be further expressed as

(2) $$SES_{x(t)}=\left|\text{FFT}{\left(x_{\text{filter}}(t)\right)}^{2}\right|$$

**Figure 4.**
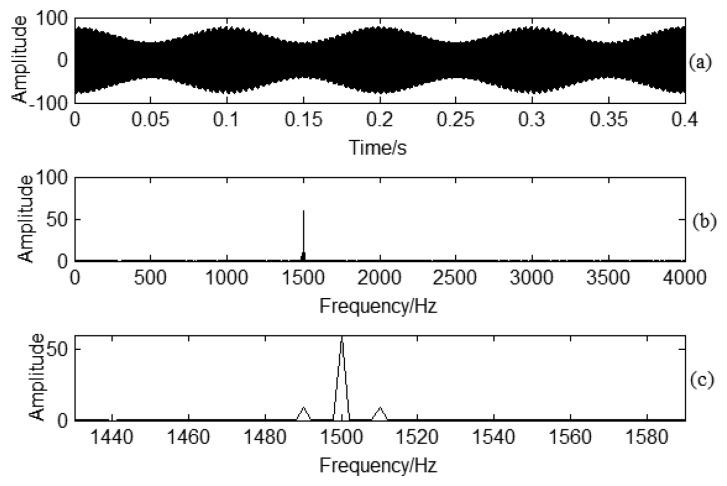
Signal demodulation analysis: (**a**) Time domain signal for the simulation signal; (**b**) Spectrum analysis for the simulation signal; (**c**) Enlarged frequency area for the carrier frequency area.

Based on Equations (1) and (2), the CF modulated to high frequency can be effectively demodulated from the original monitored signal if a suitable frequency band is applied for analysis. The second order cyclostationary signal contains much information about machine incipient fault characteristics. Therefore, SES can be used for early modulated frequency determination. When there is a crack in a blade, it will lead to different blade vibrations. The blade vibration frequency will be modulated to BPF. According to the cyclostationary signal analysis, this also belongs to the second order characteristics. Thus, amplitude modulation signal analysis and feature extraction can be investigated by using SES. For a amplitude modulation signal, *sig* (*t*) this can be expressed as Equation (3)

(3) $$\text{sig}(t)=A(1+B\cos(2\pi F_{e}t))\sin(2\pi F_{c}t)$$

where *F_c_* = 1500 *Hz*, *F_e_* = 10 *Hz*, *A* = 60, *B* = 0.3 . *F_c_*, *F_e_* correspond to carrier frequency, and modulation frequency, respectively. The corresponding sampling frequency is 10,240 Hz for the simulation signal. Based on Equation (3), an amplitude modulation signal can be obtained as shown in Figure 4a. The Fourier spectrum analysis is shown in Figure 4b. The main frequency is 1500 Hz. The modulated frequency 10 Hz can be obtained by enlarging the frequency domain around the carrier 1500 Hz frequency shown in Figure 4c. It is obvious for the sideband frequency around the carrier frequency if there is no noise interference in the signal.

**Figure 5.**
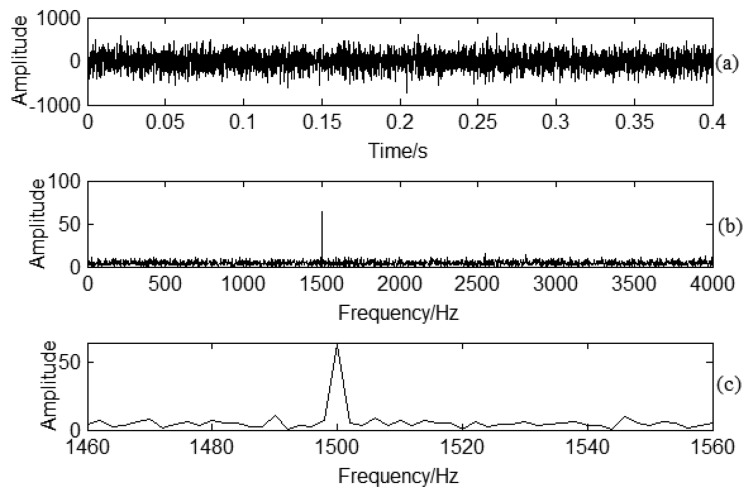
Frequency spectrum analysis: (**a**) Time domain signal for the noise interference signal; (**b**) Spectrum analysis for the noise interference signal; (**c**) Enlarged frequency area for the carrier frequency area.

Strong noise interference is added to the simulation signal as the characteristic information is usually overwhelmed by noise under practical working conditions. The obtained signal is shown in Figure 5a. In the frequency spectrum analysis, there is clear broad frequency band noise effect shown in Figure 5b. To determine the modulated signal, and enlargement of the carrier frequency area in the spectrum is shown in Figure 5c. Obviously, the enlarged frequency area is not clear due to the noise interference. The noise interference has an effect on the CF determination, therefore, it is difficult to classify the CF just according to sideband frequency spectrum analysis if there is strong noise interference.

**Figure 6.**
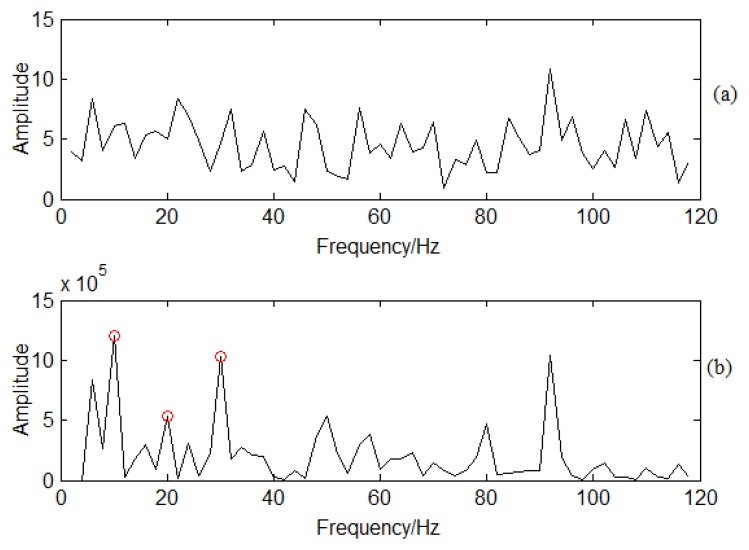
Demodulation analysis for the simulation signal: (**a**) Envelope spectrum analysis; (**b**) SES method.

Figure 6 provides the demodulation analysis for the noise interference signal. Envelope analysis is first used for feature extraction as shown in Figure 6a. The 10 Hz modulation frequency cannot be obtained as the noise interference. SES is used to demodulate the CF. The filter frequency band is 1400–1600 Hz based on the frequency scope. Figure 6b provides the spectrum analysis using the SES method. There are clear 10, 20, and 30 Hz signals. This is typical multiple frequency information. There are also other multiple frequencies, such as 50, 80, and 90 Hz. As well, the amplitudes corresponding to 50, 80, and 90 Hz are also obvious. This means that there is frequency modulation based on the spectrum analysis. The modulation frequency is 10 Hz. The modulation frequency can be obviously obtained by using SES though there is strong noise interference, proving that SES can be used for incipient CF determination from strong noise interference.

### 2.2. Blade Crack Characteristics

In general, centrifugal compressor casing vibration and radiation noise are closely related to blade BPF and its harmonics. It is also generated by the interference between rotor and stator during blade rotation. BPF has high energy in the pressure frequency spectrum. It is the main source of centrifugal compressor noise and can be applied to estimate blade health condition [6]. Its value can be determined by shaft speed multiplying the number of blade. BPF can be calculated by Equation (4)

(4) $$\text{BPF}=\frac{\text{RPM}}{60}\times N$$

where *RPM* is the shaft speed and *N* is the number of blades in the impeller.

As BPF is a high frequency component, the low frequency components such as blade vibration can be modulated to BPF during blade rotation. The modulation information will appear as the sideband frequency of the BPF. For unbalanced rotor conditions, the shaft frequency (SF) will also be modulated to the BPF, giving a sideband frequency around the BPF for unbalanced condition. Sideband frequency could be used to determine the modulated CF. This is also similar to the simulation signal analysis. As the information produced by cracks is weak and overwhelmed by strong noise, feature extraction is important to determine the CF. The sideband frequency produced for blade cracks is different from SF. It can be used to alert of a crack in a blade. It doesn't mean there is a blade with cracks if SF is the sideband frequency for BPF [6]. It is difficult to classify CF just according to the spectrum at the beginning of crack as the magnitude of the blade vibration is weak compared with the amplitude of BPF. SES is helpful to improve the recognition for blade cracks, thus, it is beneficial for early blade crack classification.

### 2.3. Blade Crack Classification Method

**Figure 7.**
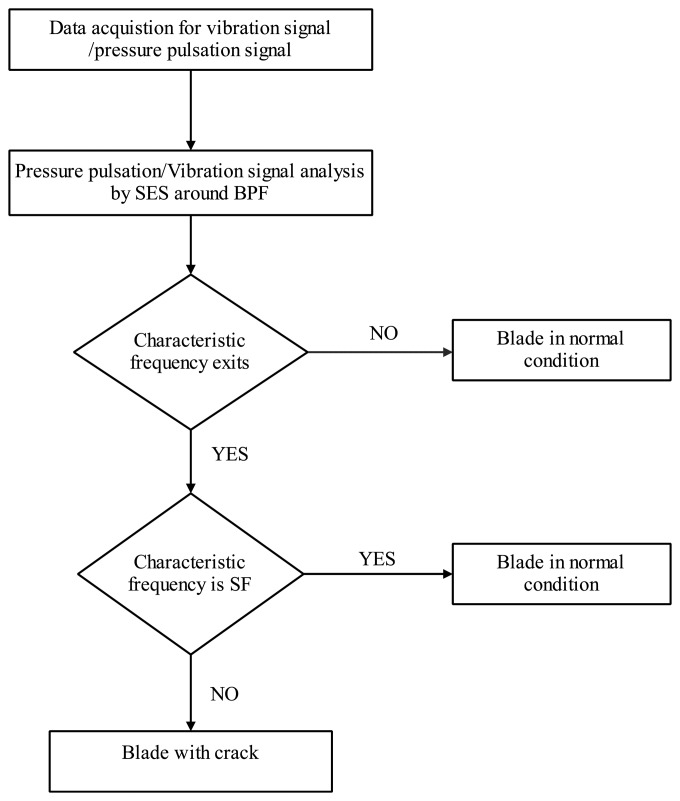
Flowchart for blade crack classification by using PP or vibration signals.

To classify blade crack working conditions, SES is applied to the casing vibration and PP signal analysis based on the blade crack signal mechanism. The key steps for characteristic frequency determination are shown as Figure 7. First, PP or the vibration signals are determined based on the best suitable position according to blade crack classification. This is also a key step to determine the crack information because the sensor location has a direct effect on classification accuracy. Then, CF for PP or vibration signal demodulation is determined by using SES. Finally, blade condition is determined by CF analysis. If the demodulated frequency is the same as SF of the rotor, it means there is not any crack in the blade. Otherwise, it means there is a crack in the blade. Inspection must be carried out to keep centrifugal compressor blade working in normal conditions.

## 3. Experimental Setup

### 3.1. Test-Rig

**Figure 8.**
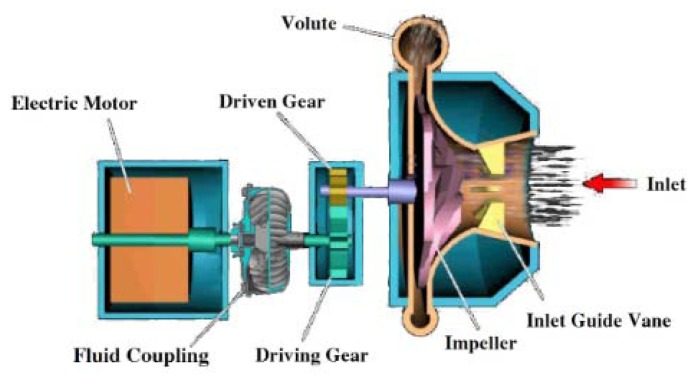
Schematic of the test-rig.

To verify the effectiveness of this method, an experiment was carried on blade crack condition classification by using SES method based on casing vibration and PP signals analysis in a test-rig. The schematic diagram for the test-rig is shown in Figure 8. It contains an electric motor, fluid coupling, gearbox and impeller. The impeller is a semi-closed one with 800 mm diameter. It is an experimental impeller for performance testing. By using fluid coupling, the rotating speed for impeller varies from 500 RPM to 9000 RPM. With the speed-up gearbox, the rotation speed of impeller can meet the designed one. The ratio between the driving and driven gears is 126/43 = 2.93. The related experimental parameters are listed in Table 1. In this experiment, the speed of the impeller is 4500 RPM for different conditions. The SF and BPF correspond to 75 Hz and 975 Hz, respectively. The experiment was carried on under two conditions, normal and crack conditions. The crack length during the experiment is 50 mm.

### 3.2. Data Acquisition

**Figure 9.**
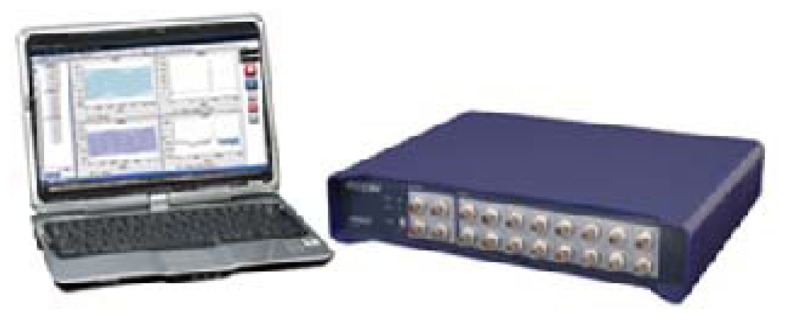
Vibration data acquisition system.

It is important to apply suitable sensors and a data acquisition system for accurate blade crack classification. The data gathering system is shown in Figure 9. It is a MI-7016 16-channel synchronous data gathering system produced by the ECON Company (Hangzhou, Zhejiang, China).

An acceleration sensor is used to monitor the vibration signal. Accelerometers produced by PCB Piezotronics (New York, NY, USA) are used in this experiment. The sensitivities are 93 mv/g and 95 mv/g. An acoustic pressure sensor is used to monitor the PP signal and its sensitivity is 41.2 mv/Pa. PP signal, vibration signal and shaft speed signal are acquired together by the MI-7016 system. As well, AE signal is also monitored by data gathering system produced by the Physical Acoustic Company (Princeton, NJ, USA). Eddy current signal is also used to monitor shaft displacement information.

**Figure 10.**
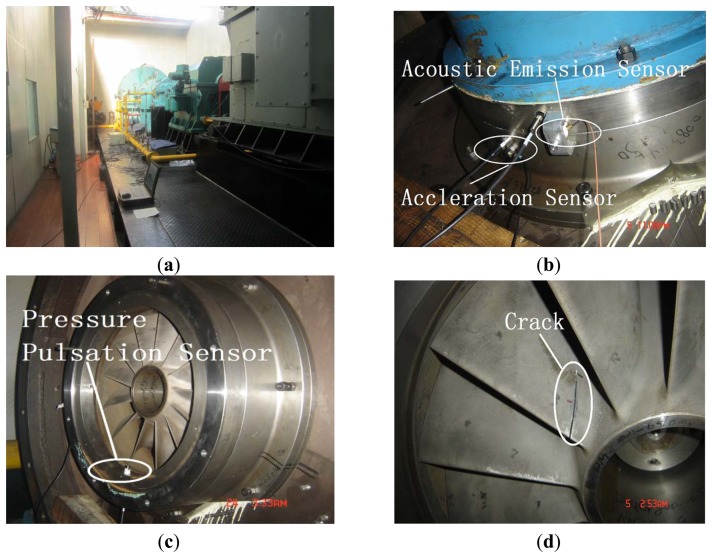
Pictures of the experiment. (**a**) Picture of test-rig; (**b**) Picture of transducer location; (**c**) Picture of blade monitoring; (**d**) Enlarged crack picture.

Figure 10a is the picture of the test-rig for these experiments. The acceleration sensor is located at the inlet casing to monitor vibration signals. It corresponds to the crack location in the axial direction. The location for the vibration sensor is important as it is directly related with the pattern recognition efficiency for the determination of weak blade crack information. If the vibration sensor position is far from the casing position corresponding to blade crack, it is difficult to classify blade cracks using vibration signal analysis [11]. The acoustic pressure sensor is installed in the inlet pipe to monitor PP shown as Figure 10b,c. AE sensors are also located at the inlet pipe as many researchers also investigate crack classification by monitoring high frequency signal variation. Figure 10d provides an enlarged picture of the processing crack in a blade. According to the practical working conditions for blade crack and failure, the crack usually generates at a position near the end of the impeller hub. This is also shown in Figure 1. In this experiment, the data sampling frequency is 48,000 Hz for vibration and PP signals.

## 4. Signal Analysis

### 4.1. Pressure Pulsation Signal Analysis

**Figure 11.**
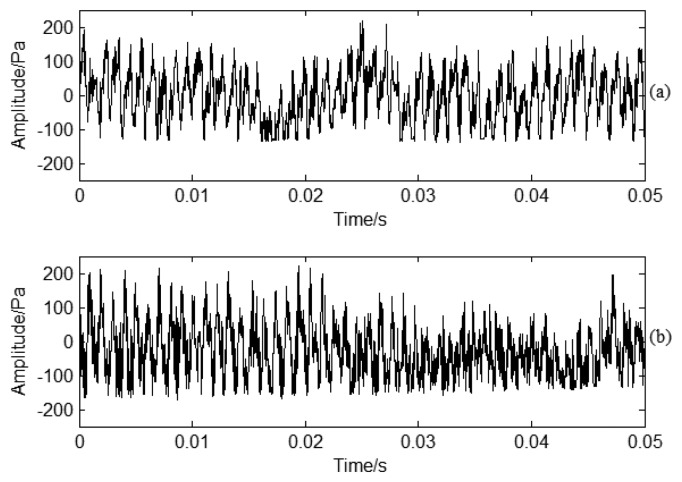
PP time domain signal analysis under different working conditions: (**a**) Normal conditions; (**b**) Crack conditions.

PP generates during impeller rotation by transferring dynamic energy to pressure energy. BPF is the main characteristic frequency for PP signals. The PP signal contains much information about the fluid dynamic characteristics. The PP sensor is installed near the blade crack circumference. It is not convenient to install the PP sensors compared with acceleration sensor, but it may be fixed during centrifugal compressor unit installation. Time domain PP signals under normal and crack conditions are shown in Figure 11a,b, respectively. There is no clear difference in time domain information. The amplitude is basically the same. Strong noise interference has a great effect on the classification process. Therefore, it is difficult to classify the crack information from normal conditions just according to time domain information. If there is a crack in a blade, blade vibration is different from normal working conditions, but as the information is weak using PP signal to monitor blade vibration indirectly, time domain signals cannot provide clear characteristics for the blade crack as the information is overwhelmed by noise.

**Figure 12.**
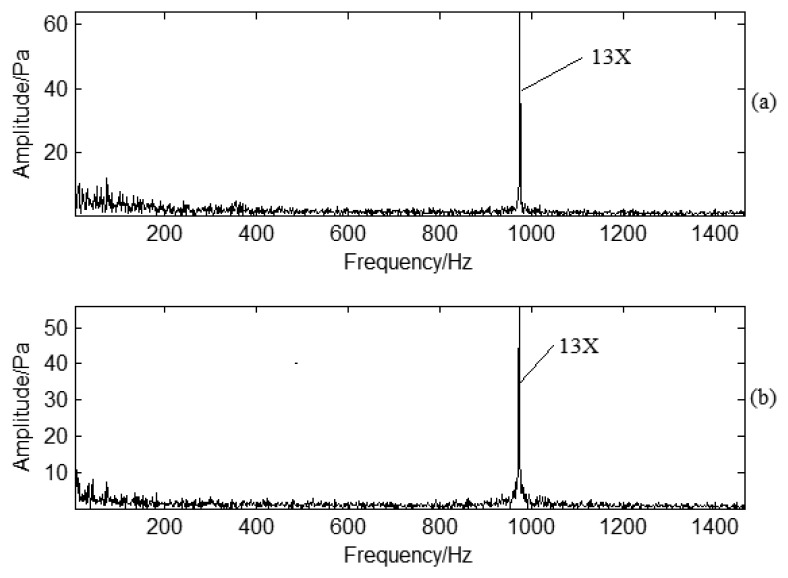
PP frequency domain signal analysis under different working conditions: (**a**) Normal conditions; (**b**) Crack conditions.

**Figure 13.**
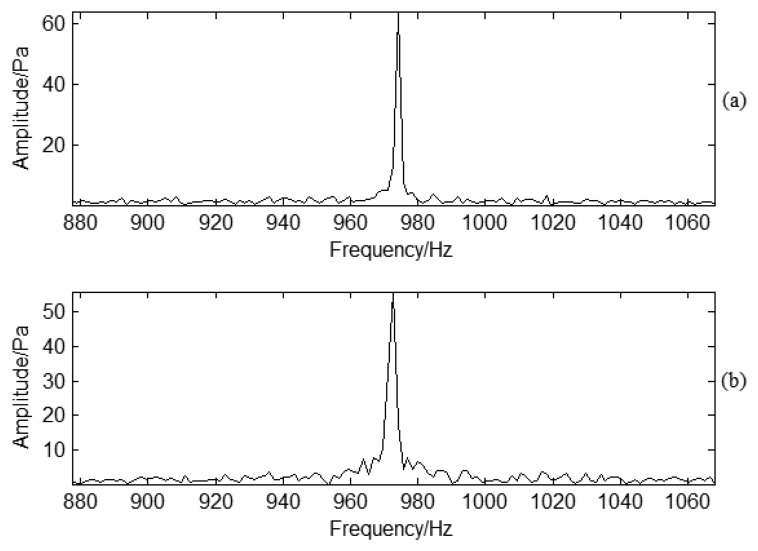
Enlarged frequency domain signal analysis under different working conditions: (**a**) Normal conditions; (**b**) Crack conditions.

To determine the characteristic information for blade cracks, frequency domain PP signals under normal and crack conditions are shown as Figure 12a,b, respectively. The waveforms are basically similar for the two conditions. BPF is typical in the spectrum analysis. There are also low frequency lines around BPF, but normal conditions and crack conditions are almost the same in the frequency spectrum analysis. It is impossible to classify the fault information based on time or frequency analysis of PP signals. To verify the effectiveness for sideband frequency analysis, the enlarged frequency spectra are shown in Figure 13a,b, respectively. It is obvious that the sideband frequency is not clear due to the noise interference. For the PP signal, the sideband frequency cannot provide clear information about the modulated frequency. This is different from Rao's experimental investigation on gas turbine blades [10] as the structure of a compressor blade is different. Based on the above analysis, it is difficult to separate normal or crack conditions just according to time or frequency information under strong noise interference conditions. Further investigation should be carried on for crack determination.

**Figure 14.**
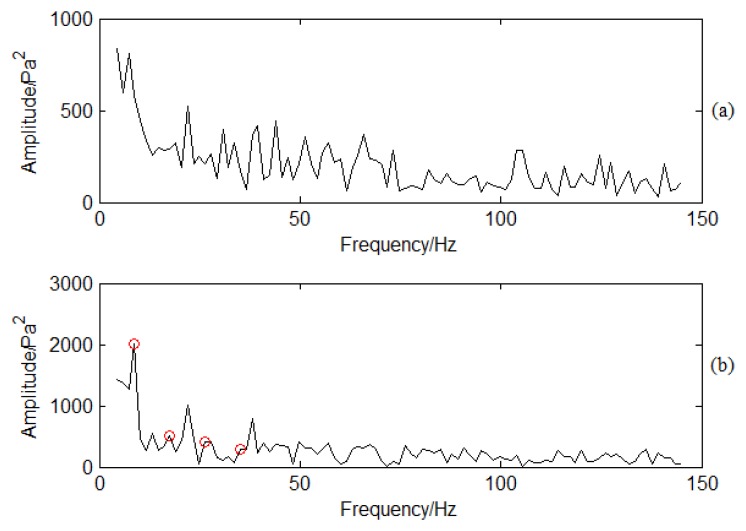
SES for PP signal under different working conditions: (**a**) Spectrum using SES for normal conditions; (**b**) Spectrum using SES for crack conditions.

SES is applied to PP signal analysis based on the flowchart shown in Figure 7 to demodulate the high frequency signal. The filter frequency band is designed between 900 Hz to 1100 Hz by containing the BPF. Spectra are determined by using SES for the normal and crack conditions shown in Figure 14. There is no obvious frequency multiplication for the normal conditions shown in Figure 14a. Frequency multiplication is a regular method to estimate whether there is fault by using envelope analysis. Therefore, it means that the blade has no cracks and is working under normal conditions. On the contrary, there is an obvious frequency multiplication for crack conditions as shown in Figure 14b. The CF is 8.8 Hz. The red circles in Figure 14b correspond to 8.8, 17.6, 26.4, and 35.2 Hz. It also means that there is typical frequency multiplication. The base frequency for the demodulated signal is 8.8 Hz. The crack vibration generates the 8.8 Hz CF. It functions on the air, so does the PP signal. The blade vibration frequency is modulated to the BPF signal. With traditional time/frequency methods it is difficult to classify it as the information is weak, but SES can demodulate the CF from the high frequency BPF. It is obvious CF can be determined compared with sideband frequency analysis. It also verifies the effectiveness of SES for crack vibration CF determination.

### 4.2. Casing Vibration Signal Analysis

**Figure 15.**
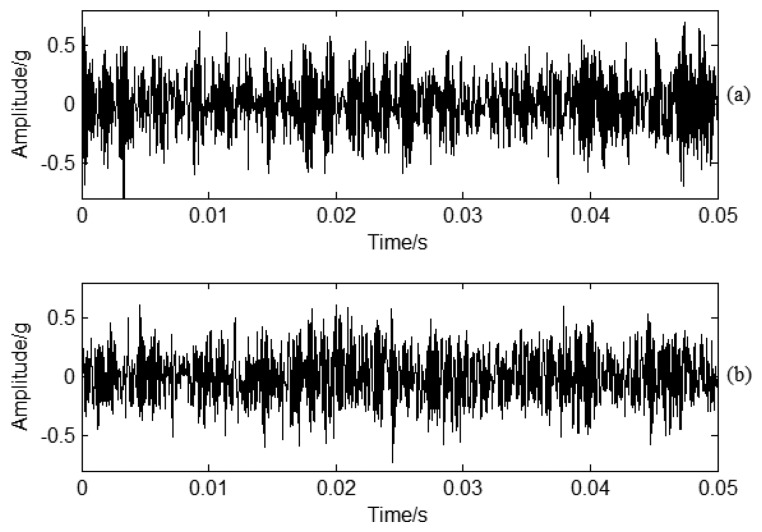
Vibration signal time domain analysis under different working conditions: (**a**) Normal conditions; (**b**) Crack conditions.

**Figure 16.**
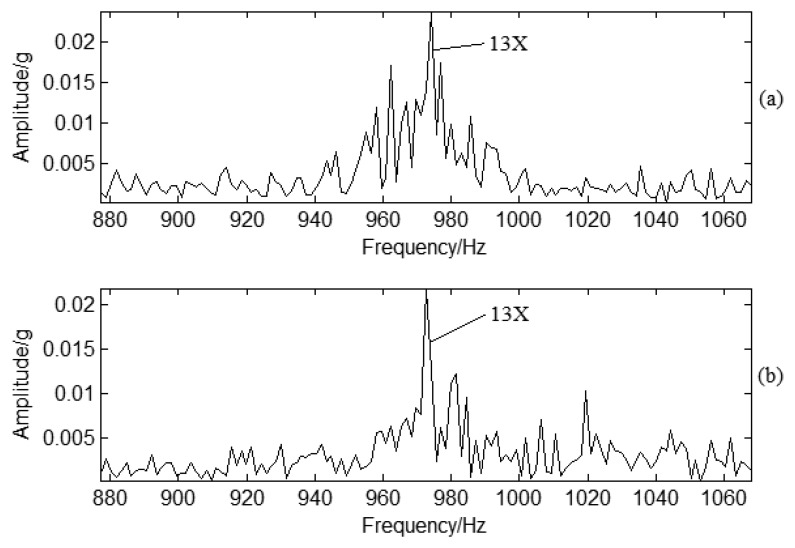
Enlarged vibration signal frequency spectrum analysis under different working conditions: (**a**) Normal conditions; (**b**) Crack conditions.

The vibration signals analysis for different working conditions was also carried out for further verification of this method. Figure 15a,b corresponds to normal and crack conditions vibration signals in the time domain. Figure 16a,b corresponds to enlarged spectrum analysis for normal and crack working conditions in the frequency domain, respectively. The BPF is also clear in the frequency domain, but it is impossible to classify CF based on the time domain or frequency domain signal analysis as there is strong noise interference. The blade vibration information is weaker compared with the PP signal. As well, the casing vibration response is also complicated compared with PP. It is impossible to determine the CF based on the sideband frequency method by enlarged spectrum analysis. For a practical centrifugal compressor or a closed loop test-rig, the complicated working process makes it impossible to determine the modulated signal just according to sideband frequency analysis. CF should be determined for better classification. Therefore, it is important to use SES for determination of further characteristics using vibration analysis.

**Figure 17.**
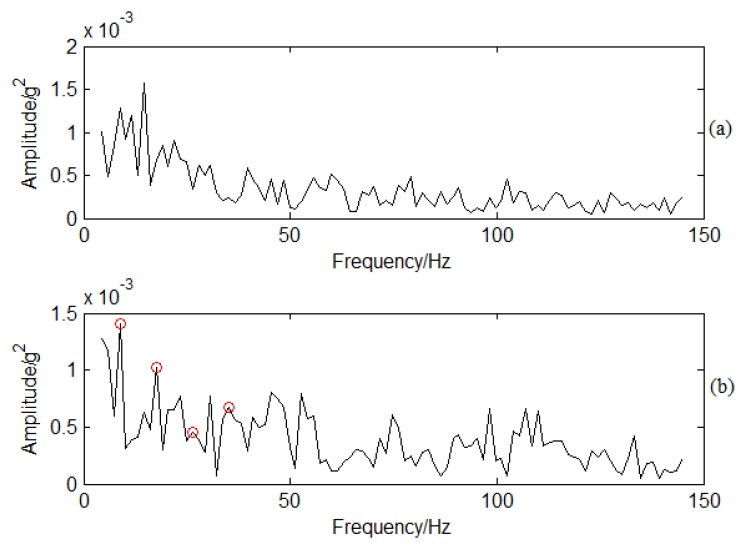
SES for vibration signal under different working conditions: (**a**) Normal conditions; (**b**) Crack conditions.

SES is applied to casing vibration signal analysis to determine the CF. The filter frequency band is also between 900 Hz to 1100 Hz, the same as in PP signal analysis. The demodulated spectrum can be obtained as shown in Figure 17. There is not any frequency multiplication for normal condition vibration signals as shown in Figure 17a, but there is a clear frequency multiplication for crack conditions as shown in Figure 17b. The red circles shown as Figure 17b correspond to 8.8, 17.6, 26.4, and 35.2 Hz. This means that there is typical frequency modulation around BPF. The base frequency for the demodulated signal is also 8.8 Hz, the same as in PP signal analysis which is also from air dynamics energy transmission and it is different from SF. The air dynamics is an input exciting force which leads to the casing vibration response. It also means blade crack information can be transferred to the centrifugal compressor casing by air. Therefore, the information can be determined by casing vibration.

### 4.3. Discussion

As blades crack, it leads to blade vibration during the centrifugal compressor high speed rotation. The blade frequency is directly related to the blade crack length. As well, it is also related to the impeller rotation speed. The blade vibration frequency will increase with the growth of blade crack with the same rotation speed. For different crack lengths, there will be different CF, therefore, it is an effective characteristic parameter for blade crack condition classification. For the early stages of a blade crack, the CF will be small and is not convenient for classification. Based on the fact the blade vibration frequency can be modulated to BPF, the PP and vibration signals can be used to determine the crack CF as the crack vibration data is not acquired directly. The SES method is much better compared with sideband frequency analysis. The CF can be determined by vibration signals or PP signals based on blade crack feature extraction. This doesn't mean one sensor is enough for the condition monitoring and recognition though the CF is the same. Vibration signals are convenient to monitor compared with PP signals, but they are also easily disturbed by other vibration sources and noise. As well, a suitable sensor position also affects the accuracy of classification. At the early stages of a crack, the CF for the blade crack is weak. It is better to classify blade cracks with two-kind sensors together. Multi-sensor fusion is an effective tool for machine condition classification [2].

## 5. Conclusions

In this research, vibration and PP signals are used for blade crack condition monitoring and classification using the squared envelope spectrum method. Experiments on an industrial centrifugal compressor with a cracked blade were carried out to verify the effectiveness of this method. CF of blade crack information can be obtained by using SES demodulation. Vibration signals and PP signals have the same modulation frequency. It is verified that crack characteristics can be classified by using PP or vibration signals. Further investigations will be carried on multi-fault condition classification.

## Figures and Tables

**Table 1.** Characteristic parameters for φ800 test-rig.

**Speed (RPM)**	**4500**
Number of blades	13
Shaft frequency (Hz)	75
Blade passing frequency (Hz)	975
